# Polarization independent silicon micro antenna based on a subwavelength metamaterial

**DOI:** 10.1038/s41598-025-97833-3

**Published:** 2025-04-17

**Authors:** Sarra Salhi, Xiaochen Xin, Daniel Benedikovič, Carlos Alonso-Ramos, Laurent Vivien, Delphine Marris-Morini, Eric Cassan, Winnie N. Ye, Daniele Melati

**Affiliations:** 1 Centre de Nanosciences et de Nanotechnologies, Université Paris-Saclay, CNRS, 91120 Palaiseau, France; 2 Department of Electronics, Carleton University, Ottawa, ON K1S 5B6 Canada; 3 Present Address: Department Multimedia and Information-Communication Technology, University of Zilina, 01026 Zilina, Slovakia

**Keywords:** Integrated optics, Metamaterials, Silicon photonics

## Abstract

Optical antennas are key components of an optical phased array system, enabling light coupling between the chip and the free space. In such systems, surface gratings are commonly used as antenna elements, which however suffer from a strong polarization sensitivity of their scattering angle and efficiency. Here, we propose a versatile approach to realize micro antennas based on surface gratings with a polarization insensitive behavior exploiting a subwavelength metamaterial in the silicon-on-insulator platform. In the experimental demonstration, the antenna successfully achieves the same diffraction angle of 10° for both TE and TM polarizations and an estimated scattering efficiency of -4 dB despite a very compact footprint of 6.4 $\mu m$ x 2.9 $\mu m$. The difference in diffraction efficiency between the two polarizations remains smaller than 1 dB over a bandwidth of 31 nm.

## Introduction

Optical Phased Arrays (OPAs) for on-chip beam forming and steering represent a promising alternative to traditional mechanically moving components in various applications, notably in light detection and ranging (LiDAR) and free-space optical communications^1–5^. Integrated optical antennas, which are responsible for emitting and receiving light to and from free space, are the fundamental components of an OPA. Although recent literature demonstrated optical antennas based on metallic structures, dielectric pillars, fiber lenses, photonic crystals and ceramics^6–9^, dielectric surface gratings are still the most popular choice for OPAs because of their relative ease of fabrication and the possibility of achieving high efficiency^10–14^. Surface gratings with high diffraction performances for either transverse-electric (TE) or transverse-magnetic (TM) waveguide modes have been extensively reported in the literature^15–26^, especially exploiting L-shaped diffraction elements to break the vertical symmetry and achieve high directionality^18,27–30^. Grating antennas with specially designed features such as an optimized radiation pattern, improved steerability, and reduced grating lobes have been reported as well^31–34^. Micron-scale antennas are of particular interest for OPAs because the reduced footprint and broad radiated beam are fundamental to achieve large steering ranges^35^. The main drawback of surface gratings is their strong polarization sensitivity, arising from the intrinsic birefringence of the grating Bloch mode which causes TE and TM polarized light to be diffracted at different angles and with different efficiencies^36,37^. The dependence of the optical behavior of the antenna on the polarization of the light can represent a significant challenge, for example in implementing polarization division multiplexing in optical communication systems or ensuring consistent and reliable coupling efficiencies of receiver devices. Polarization sensitivity is particularly pronounced in the high contrast, silicon-on-insulator (SOI) platform commonly used for the development of OPAs because of its strong waveguide birefringence. Nonetheless, optical antennas with a single feeding waveguide capable of effectively supporting both TE and TM polarizations would be extremely beneficial for enhancing the robustness and performance of OPA systems.

A polarization insensitive grating requires the TE and TM polarized Bloch modes to have the same effective index, a condition that brings to designs with low efficiencies in the 220-nm SOI platform^38^. A recent design of a grating coupler for standard fiber coupling reported in^39^ suggests (based only on simulations) that a considerable improvement could be achieved in the efficiency moving to a thicker silicon layer (500 nm) which allows to better guide the TM waveguide mode. Alternatively, subwavelength grating metamaterials (SWG) have also been explored in the literature as a promising path to increase the degrees of freedom available for the design of the grating and achieve a polarization insensitive behavior with higher scattering efficiencies^40–43^. Similarly to metasurface^44–46^SWGs are realized by arranging their unit cells at a distance much smaller than the wavelength, resulting in an effective artificial material whose optical properties can be controlled by modifying the geometry of the unit cells^47^. For example, a grating coupler for standard fiber coupling with a difference between TE and TM coupling efficiencies smaller than 1 dB over a bandwidth of 12 nm was experimentally demonstrated in^40^. However, the grating was based on a suspended membrane silicon waveguide that required removal of the buried oxide and made it difficult to implement the device using standard silicon photonics processes.

In this work, we report on an innovative approach to design polarization-insensitive micro antennas targeting free-space coupling and OPA applications. The antennas exploit surface gratings that are compatible with standard fabrication processes for silicon photonics. The antenna does not require the removal of the buried oxide and can be implemented with an oxide top cladding. The simultaneous use of an SWG metamaterial and L-shaped grating elements allows achieving the same scattering angle for TE and TM polarized light as well as a high scattering efficiency. In the proof-of-concept experimental demonstration reported here, the antenna has a minimum feature size of 120 nm and a very compact footprint of 6.4 $\mu m$ x 2.9 $\mu m$ (roughly 10 to 15 times smaller than common surface gratings designed for standard fiber coupling). A 500-nm SOI platform is chosen to ensure the best antenna performance but this does not compromises the generality of the device concept, which could also be implemented in a 300 nm platform, as discussed in section 2.1. 3D FDTD simulations show that both TE and TM polarized modes are diffracted with the same scattering angle of 10$^{\circ }$ and that, despite the compact size, the upward diffraction efficiency is as high as -2.8 dB and -2.4 dB for the two polarizations. The experimental characterization of the antenna is in a very good agreement with simulations and demonstrates a difference in TE-TM coupling efficiency at 10$^{\circ }$ smaller than 1 dB over a 31 nm wavelength range, with an estimated free-space upward scattering efficiency of -4 dB.

## Design of the surface grating antenna

### Antenna optimization

**Fig. 1:**
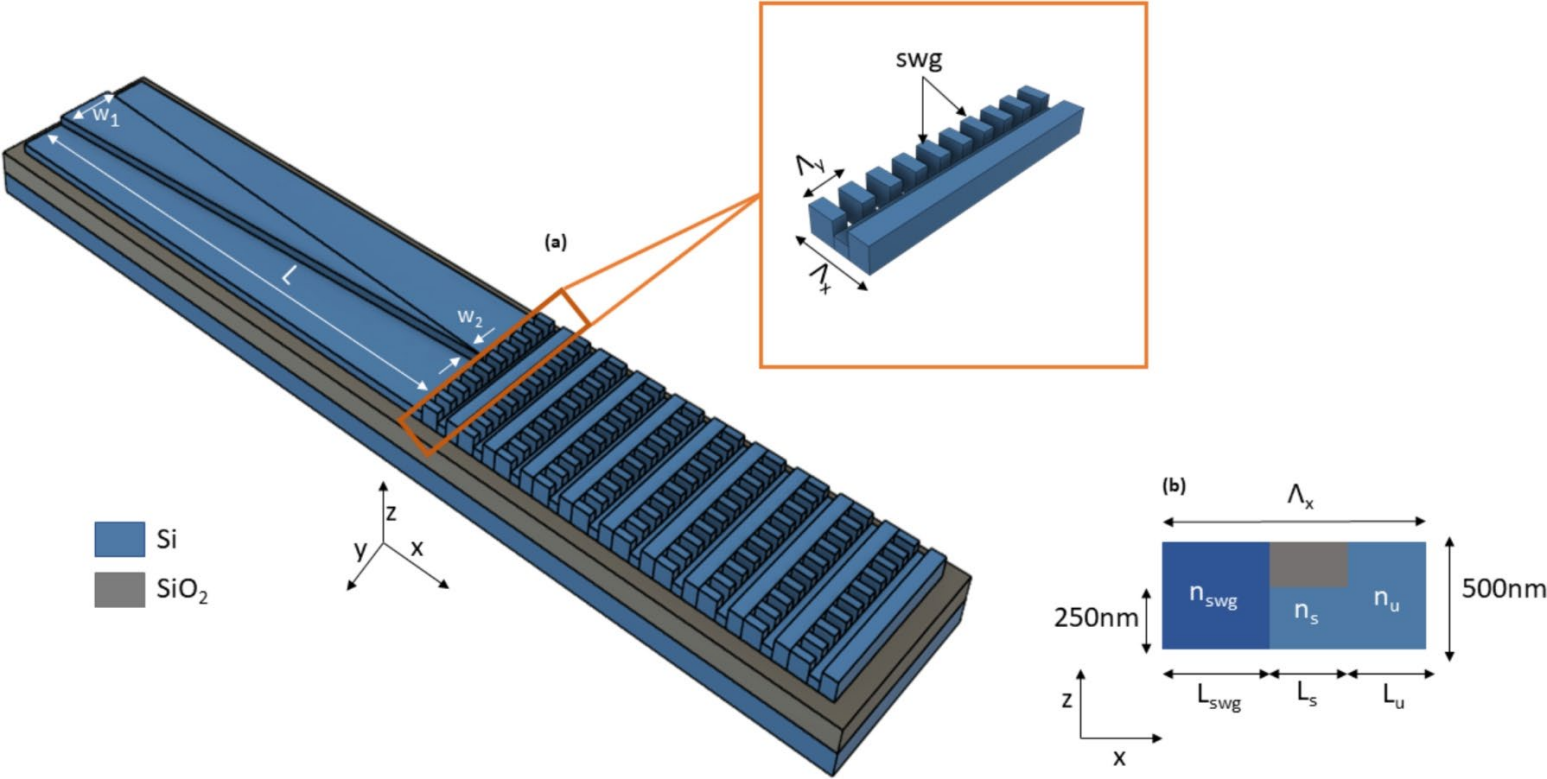
Schematic of the micro antenna. (**a**) Perspective view and (**b**) cross-section.

The diffraction angle of a surface grating can be calculated through the grating equation^42^:

1 $$\begin{aligned} \sin (\theta ) = \frac{n_B}{n_C} + m\frac{\lambda }{n_C\Lambda _x} \end{aligned}$$

where $n_B$ is the effective index of the Bloch mode of the grating, $n_C$ is the refractive index of the cladding material, $\Lambda _x$ is the period of the grating (in the propagation direction x, as defined in Figure 1 (a)) and $\lambda$ is the wavelength, 1550 nm for the design considered here. In order for the TE and TM modes to be diffracted with the same angle, resulting in a polarization insensitive grating, it is required that $n_B$ be the same for the two polarizations. We achieve this objective by incorporating an SWG metamaterial into the grating unit cell to engineer the birefringence of the grating waveguide^48^. The grating structure, as schematically represented in Figure 1 (a), includes two sections. The first one is the SWG metamaterial of length of $L_{\text {SWG}}$, with a transverse period in the y direction of $\Lambda _y$ and fill factor ff$_y$. By adjusting the value of ff$_y$, it is possible to control the equivalent effective refractive index and the birefringence of the metamaterial, as represented in the cross-sectional view of Figure 1 (b). The second section is an L-shaped geometry, partially etched to 50% of the waveguide height for a length of L$_s$ and with an un-etched part of length $L_u$. The use of an L-shaped geometry is the key to achieve strong directionality and high upward diffraction efficiency^27^. The grating has a total period $\Lambda _x$ = L$_{\textrm{SWG}}$ + L$_s$ + L$_u$ in the propagation direction x. Accordingly, the effective refractive index of the grating Bloch mode can be approximately computed as:

2 $$\begin{aligned} n_B= (n_{\textrm{SWG}}\frac{L_{\textrm{SWG}}}{\Lambda _x}) + (n_s\frac{L_s}{\Lambda _x})+ (n_u\frac{L_u}{\Lambda _x}), \end{aligned}$$

where $n_{\textrm{SWG}}$, $n_s$ and $n_u$ are the effective indices of the fundamental 1-D modes that are supported in the corresponding sections of the unit cell for the polarization of interest, as shown in Figure 1(b).These effective indices are calculated one by one using a mode solver and considering each section infinite in both the x and y directions. Silicon is used as core materials for the calculation of $n_s$ and $n_u$ . For the SWG section, the equivalent refractive index of the core material (which characterizes the optical behavior of the SWG metamaterial) can calculated from the pitch $\Lambda _y$=300 nm and duty cycle ff$_y$ of the SWG using the model described in^49^.

**Fig. 2:**
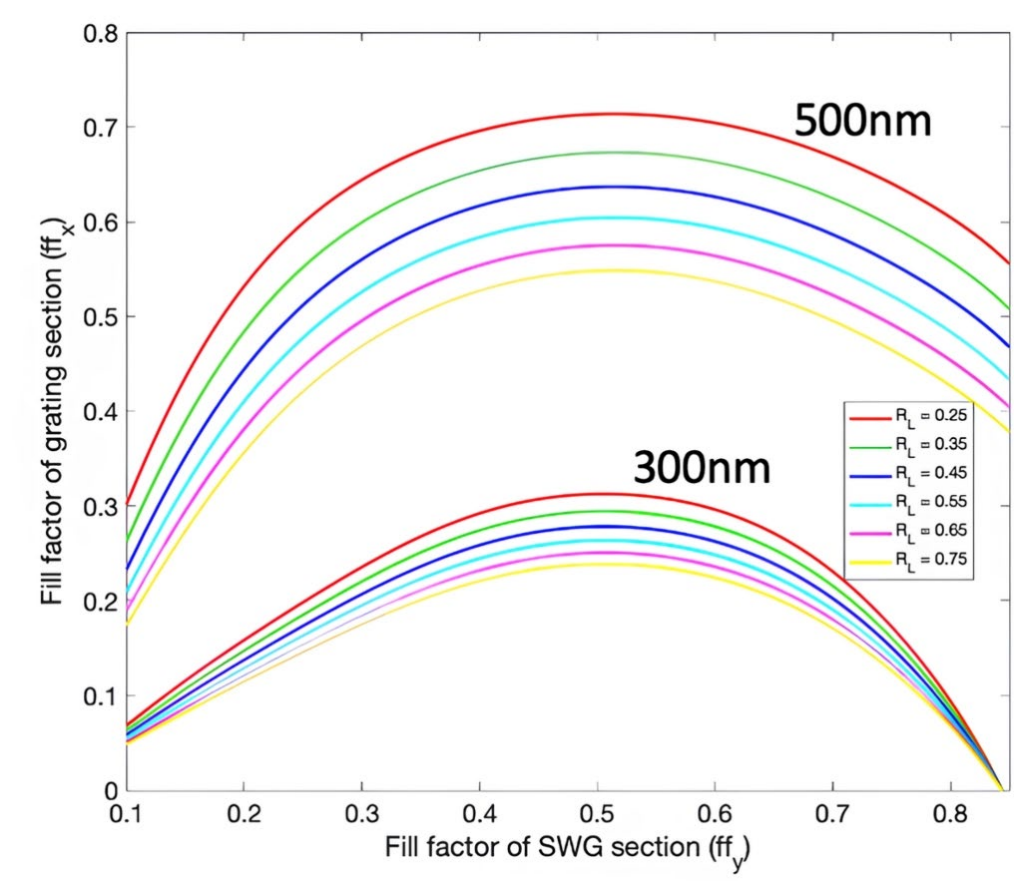
Combinations of ff$_x$ and ff$_y$ fill factors corresponding to polarization-insensitive antenna designs with different R$_L$ for 300 nm and 500 nm silicon thicknesses, R$_L$ and ff$_x$ are defined in Equations (3) and (4).

In order to optimize the performance of the antenna, we define two convenient parameters:

3 $$\begin{aligned} R_L&= \frac{L_s}{L_s+ L_u} \end{aligned}$$

4 $$\begin{aligned} \textrm{ff}_x&= \frac{L_s+L_u}{\Lambda _x}, \end{aligned}$$

being R$_L$ the fill factor of the L-shaped geometry only and ff$_x$ the fill factor of the entire unit cell of the grating. We then exploited Equation (2) to estimate the Bloch effective indices of both TE and TM modes. For each value of R$_L$, we computed the grating fill factor ff$_x$ which is needed to obtain n$_B^{\textrm{TE}}$ = n$_B^{\textrm{TM}}$ as a function of the SWG fill factor ff$_y$. With this approach, we obtained the curves shown in Figure 2 which represent a set of polarization insensitive designs for the grating. The results are reported for a silicon thickness of both 300 nm and 500 nm. The curves for a silicon thickness of 220 nm are not shown since not only the curves for different values are very close to each other, but all the points on the curves have ff$_x$ smaller than 0.1. This means that for a typical grating pitch in the range of a couple hundred nanometers, the smallest feature size of the L-shaped region is below 100 nm, which complicates fabrication.

**Fig. 3:**
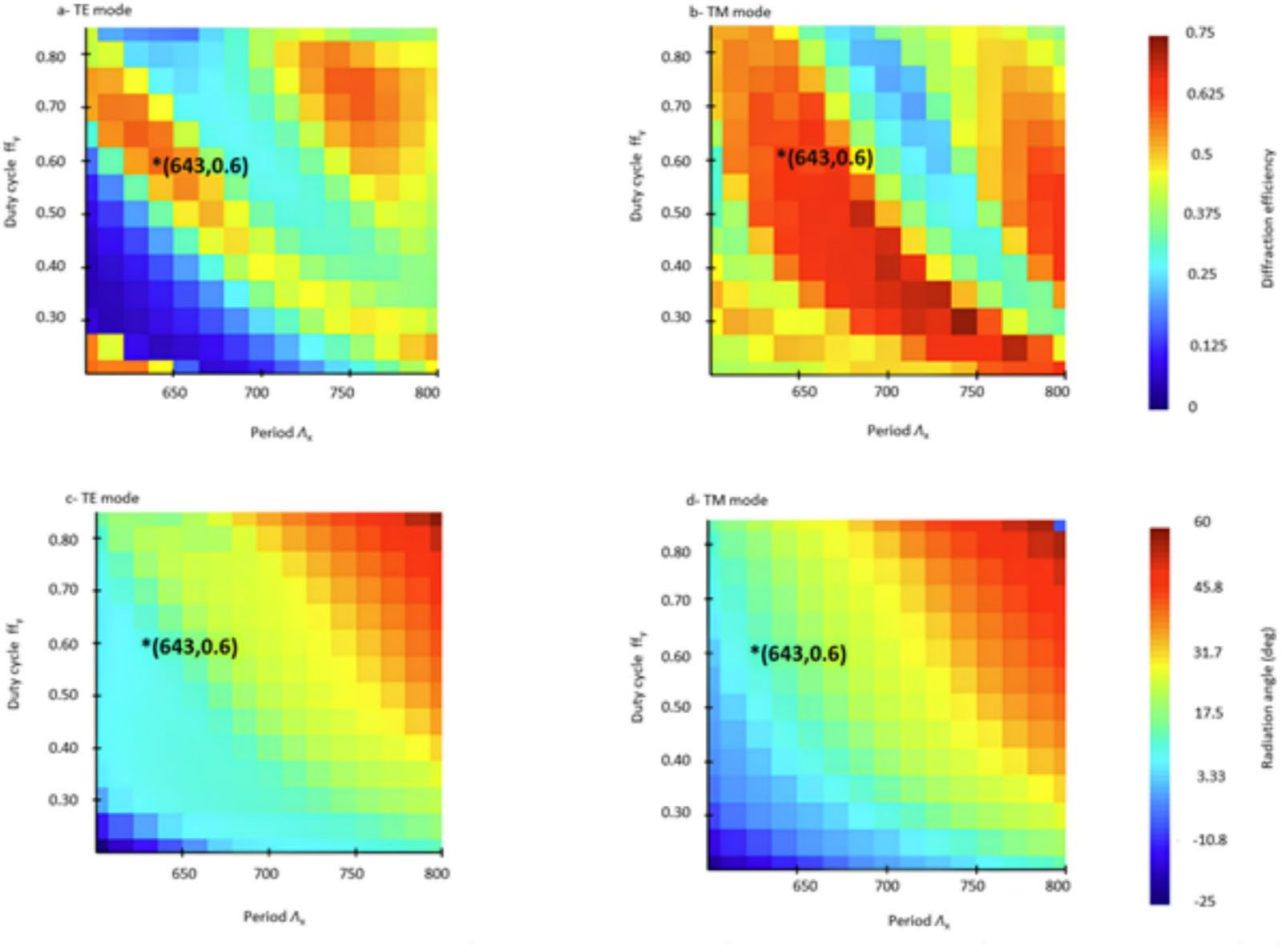
Diffraction efficiency of (**a**) TE mode, (**b**) TM mode, and radiation angle for (**c**) TE mode, and (**d**) TM mode with $R_L$=0.45.

**Fig. 4:**
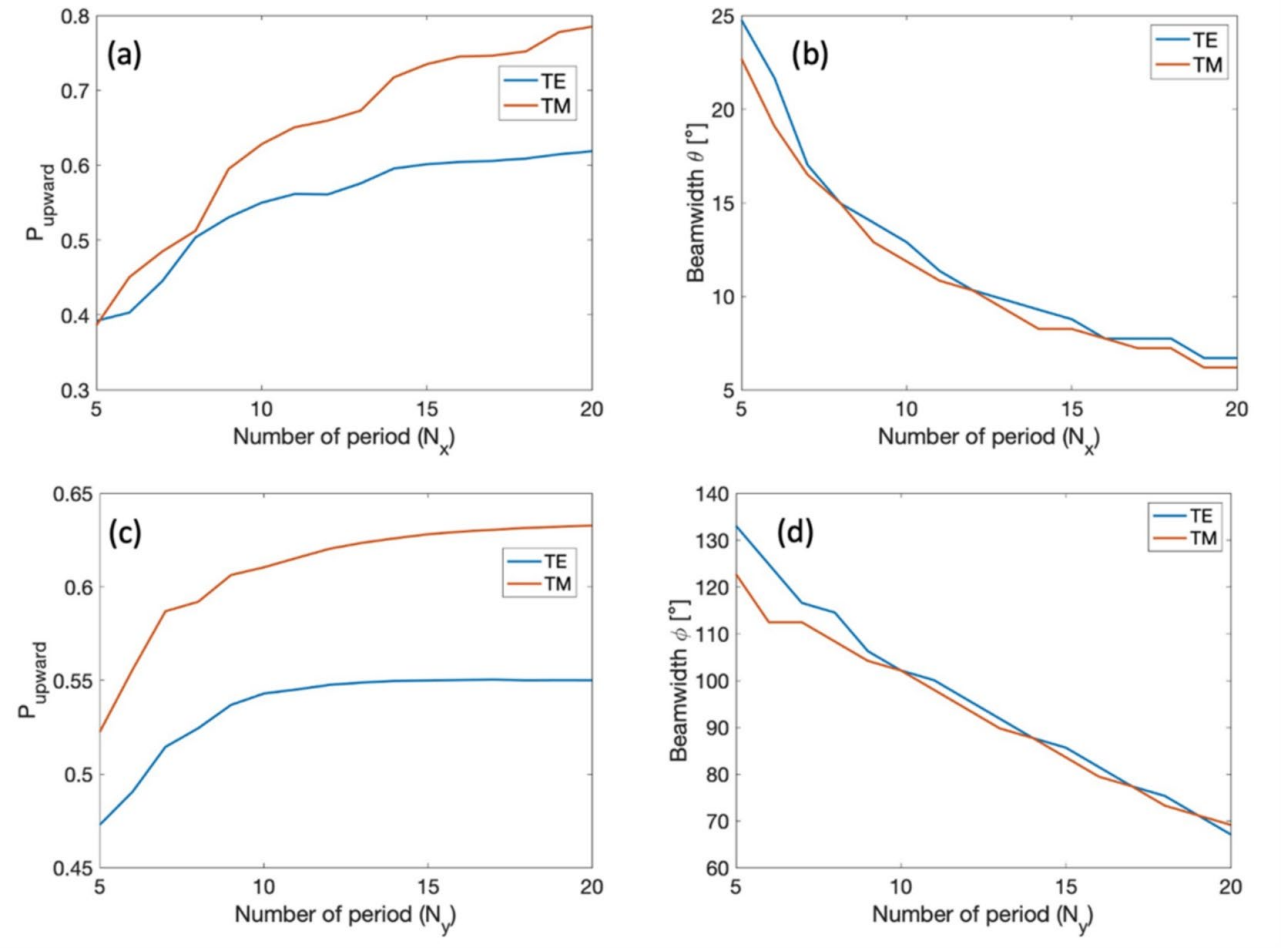
Optimization of the antenna footprint. (**a**, **c**) $P_{\text {upward}}$ as a function of the number of periods in the x (N$_x$) and y (N$_y$) directions. (**b**, **d**) Beamwidth in the elevation direction $\theta$ and the azimuth direction $\phi$ as a function of $N_x$ and $N_y$.

As detailed in the Supplementary material document, a preliminary analysis of $P_{\text {upward}}$ shows comparable results for the TE polarized mode between the two platforms, while the 500 nm silicon thickness has noticeable better performance over the 300 nm silicon for TM polarization. This is caused by the lower confinement of TM polarized light for thinner waveguides, which results in less interaction with waveguide perturbations. It was also shown in^22^ that it is difficult for thin grating structures to radiate large amount of TM polarized light. The fact that the performance of the grating for TE polarized light hardly further improves aligns with the observation made in^16^. As a result, 500 nm silicon is chosen as the platform for further optimizations. We use 3D FDTD simulations to compute the upward diffraction efficiency and diffraction angle for both polarizations for different design possibilities. We swept the parameter $R_L$ within the 0.40 - 0.55 range, $\Lambda _x$ between 600 nm and 800 nm, and ff$_y$ between 0.2 and 0.9. The corresponding value of ff$_x$ can be retrieved using Figure 2. We consider a buried oxide thickness of 3 $\mu m$, a thickness of the upper cladding of 2.2 $\mu m$ and a period of the SWG metamaterial $\Lambda _y$ = 300 nm. Figure 3 reports the calculated $P_{\text {upward}}$ and diffraction angle for $R_L$ = 0.45. The complete simulation results can be found in the material document. The marker on the color maps points to the selected design $R_L$ = 0.45, ff$_y$ = 0.6, ff$_x$ =0.62 and $\Lambda _x$ = 643nm which provides the same radiation angle 10$^{\circ }$ and diffraction efficiency around 55$\%$ for both polarizations. In this design, the smallest feature is represented by the etched sections in the SWG metamaterial, with a size of 120 nm. In addition to $P_{\text {upward}}$, one of the most important figures of merits of an antenna is its footprint, which puts constraints on its array integration and directly determines the beamwidth of the far-field (Fraunhofer) pattern through the antenna effective aperture size^50^. The optimization of the antenna’s overall length and width involves adjusting the number of diffraction periods in both the x and y directions ($N_x$,$N_y$) to investigate their impact on the far-field beamwidth and the upward diffraction efficiency ($P_{\text {upward}}$). Figure 4 shows the results of the 3D FDTD simulations. As expected, increasing the number of periods in the propagation direction N$_x$ improves the diffraction efficiency up to the point where no undiffracted light remains in the grating. Likewise, a larger antenna (larger number of transversal SWG periods N$_y$) improves $P_{\text {upward}}$ by providing a better mode confinement until the effect of the grating lateral sidewalls becomes negligible. Conversely, a larger antenna causes a reduction of the beamwidth in the far field for both azimuth $\phi$ and elevation $\theta$ direction. We chose $N_x$= 10 and $N_y$= 9 as the optimal number of periods, ensuring a compact antenna footprint of 6.4 $\mu m$ x 2.9 $\mu m$ with a limited penalty on its efficiency.

**Fig. 5:**
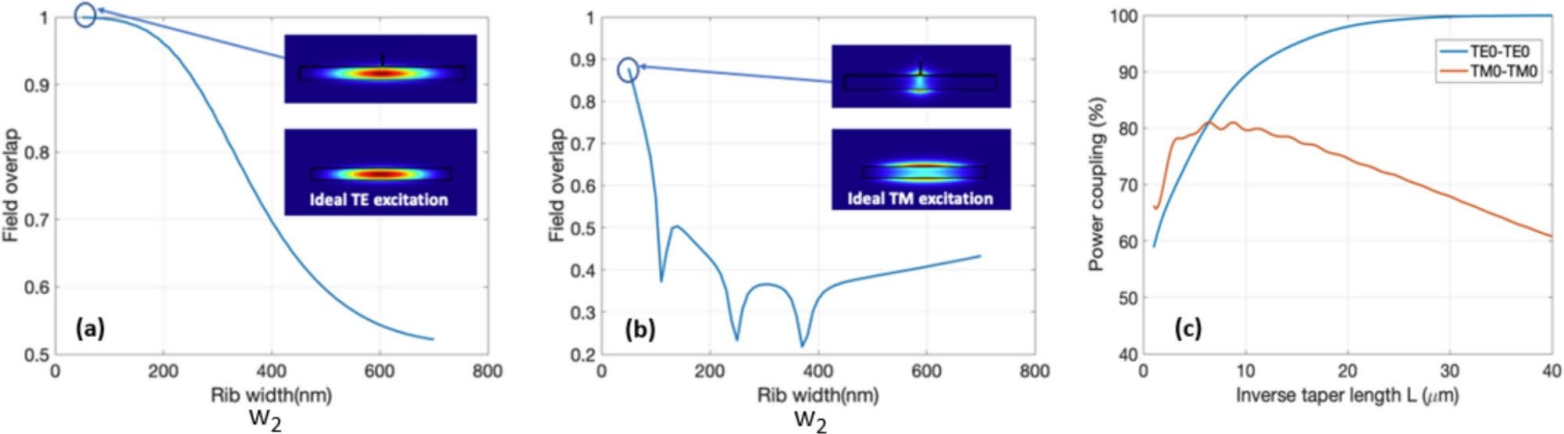
Overlap between the rib waveguide mode and the ideal grating excitation field for (**a**) the TE and (**b**) the TM fundamental modes. (**c**) Power coupling between the input and the output of the injection waveguide as a function of the inverse taper length.

### Optimization of the injection waveguide

A rib waveguide with an etched depth of 250 nm is used as the input waveguide for the grating because this solution has been proven to effectively reduce back reflection at the grating interface^15^. However, the optimized grating antenna proposed here has an overall width of 2.9 $\mu m$ and it is not practical to have a 2.9 $\mu m$-wide input waveguide while maintaining single mode operation since higher order transverse mode can easily be excited in practice. Therefore, we designed a rib-waveguide-based inverse taper as the input stage of the antenna to connect it with rest of the components on the circuit, as shown in Figure 1(a). Initially, the guided modes are confined in the rib region. As the rib becomes thinner, modes are pushed downward into the slab and then injected into the antenna. The initial width of the inverse taper $w_1$ needs to be properly designed such that only the fundamental TE and TM mode are guided. For the modes to be effectively guided in a rib waveguide, the mode effective indices must be greater than the effective index of the slab TE$_0$ mode^51^. For this reason, an $w_1$ of 500 nm is chosen. The final width $w_2$, on the other hand, has to be chosen to make sure that, at the end of the inverse taper, the modes in the rib waveguide have the best possible overlap with the ideal field excitations of the grating for both polarizations. The ideal excitation fields are defined as the TE$_0$ and TM$_0$ mode fields of a 2.9 $\mu m$ wide by 250 nm thick silicon waveguide on an SOI platform with oxide upper cladding. Field overlaps for different rib width $w_2$ for TE and TM are plotted respectively in Figure 5 (a) and (b). As expected, the best overlap occurs with small rib width. $w_2$ of 50 nm is chosen as a good compromise between ease of fabrication and device performance. For comparison, the obtained major electric field component for the two polarizations is plot in the inset alongside with the ideal excitation. Finally, the length of the inverse taper L is optimized such that as much as power is coupled into the desired modes while the length is kept as short as possible. Simulations of power coupling at different taper lengths are performed by extracting the corresponding S-parameter using an Eigen Mode Expansion (EME) solver and the results are shown in Figure 5 (c). Taper efficiency for the TM mode is limited by substrate leakage that substantially increase losses for longer tapers. As a trade-off, a length of L = 11.8 $\mu m$ is hence selected, resulting in estimated power couplings of 92$\%$ and 80 $\%$ for TE0 -TE0 and TM0 -TM0 transitions respectively. It should be noticed that a rib waveguide structure is only used here for efficient light injection in the antenna. A transition to a fully etched wired waveguide^52^ could be used to ensure a better confinement and lower bend losses for general waveguide routing.

### 3D FDTD simulation results

**Fig. 6:**
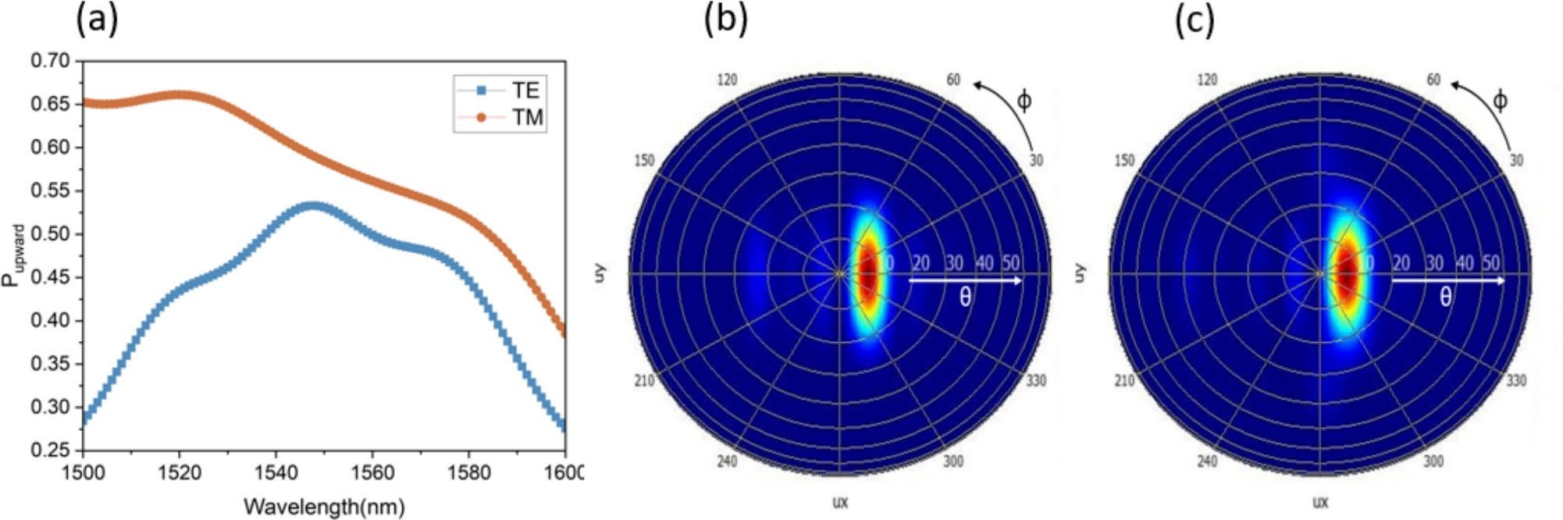
(**a**) Simulated scattering efficiency for both TE and TM polarizations. (**b**) Farfield radiation pattern for the TE and (**c**) the TM modes.

Before proceeding to fabrication, the overall performance of the antenna (including the injection waveguide) were verified using 3D FDTD simulation centered at the desired wavelength of $\lambda$= 1550 nm. Simulation results for the upward diffraction efficiency are shown in Figure 6 (a). As can be seen, the antenna achieves a similar scattering efficiency $P_{\text {upward}}$ of $53\%$ (-2.8 dB) and $58\%$ (-2.4 dB) for TE and TM modes. Since the grating design was the result of a compromise between TE and TM performance at the wavelength of 1550 nm, the antenna achieves even better TM scattering efficiency above $65\%$ for shorter wavelengths. Moreover, as shown by the far field projection of the scattered field in Figure 6 (b) and (c), the grating scattering occurs at an angle of 10$^{\circ }$ for both polarizations, confirming the polarization insensitive operation. The beamwidth is also the same for both polarizations, being equal to 9$^{\circ }$ in the elevation direction $\theta$ and 104$^{\circ }$ in the azimuth direction $\phi$. These findings are also summarized in Table 1.

**Table 1:** Simulated performance of the designed grating antenna.

	TE	TM
Diffraction angle	10$^{\circ }$	10$^{\circ }$
Upward diffraction efficiency	53$\%$ (-2.8 dB)	58$\%$ (-2.4 dB)
Beamwidth ($\theta$ x $\phi$ )	9$^{\circ }$x104$^{\circ }$	9.5$^{\circ }$x104$^{\circ }$

## Grating antenna fabrication and experimental results

In order to experimentally test the performance of our design, the grating antenna was fabricated on a SOI wafer with a silicon layer thickness of 500 nm and a buried oxide layer thickness of 3 $\mu m$. The fabrication process was completed in two steps. Firstly, the SWG metamaterial was created by electron beam lithography, followed by induced coupled plasma (ICP), resulting in a 500 nm etch depth. Next, the partial etched regions in the grating and the rib waveguide were formed using electron beam lithography and ICP etching, achieving a 250 nm etch depth. Finally, we deposited PMMA as waveguide cladding. Following the spin-coating step, the PMMA cladding was cured by gradually increasing the temperature from 40°C to 180°C over a period of 3 minutes. PPMA was chosen because it has a refractive index similar to that of $SiO_2$ ($n_{\text {PMMA}}$=1.49) and allowed to speed up fabrication within our cleanroom but a regular oxide cladding could also be used with no changes to the design.

**Fig. 7:**
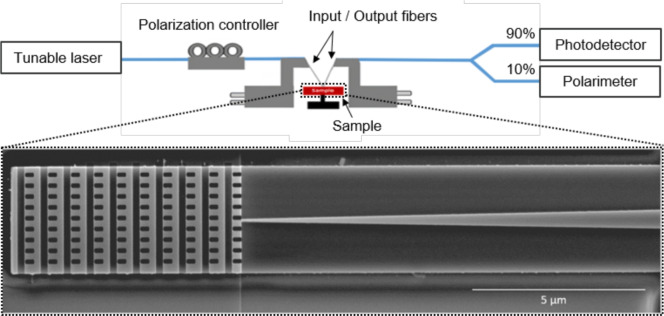
Schematic representation of the experimental setup. In the inset, scanning electron microscopy top view picture of the fabricated grating antenna.

A scanning electron microscopy (SEM) image of the fabricated antenna is shown in the inset of Figure 7. Two antennas were placed in a back-to-back configuration, connected by a rib waveguide with a length of 5 mm. To facilitate the optical characterization, the scattering efficiency of the antenna was measured using two standard single mode fibers even if the design optimization was performed for free-space coupling. The characterization setup is schematically shown in Figure 7. It includes a tunable laser and a polarization controller to set the polarization of the light injected in the grating by the first fiber. 90% of the light collected by the output fiber is sent to the detector, while the remaining 10% is fed into a polarimeter. Both input and output fibers have a 10$^{\circ }$ angle to match the designed emission angle of the antenna.

**Fig. 8:**
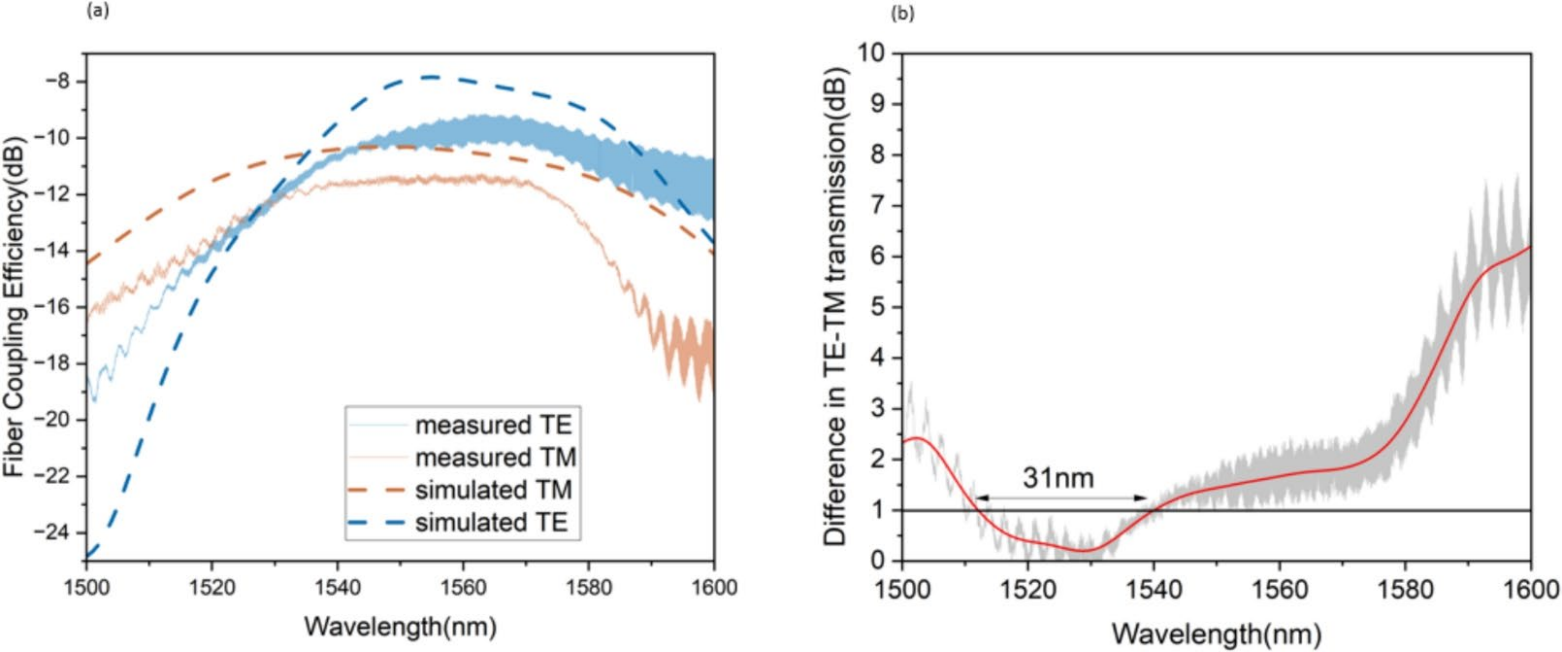
Experimental performance of the grating antenna. (**a**) Measured (solid lines) and simulated (dashed lines) fiber coupling efficiency for both TE and TM polarizations as a function of the wavelength. (**b**) Difference in TE-TM measured fiber coupling efficiency.

First, light polarization was set to TE using a reference grating with a polarization-dependent transmission realized on the same chip. We then characterized the designed antenna, obtaining the transmission spectrum shown in Figure 8 (a) with a solid blue line. The transmission was normalized to account for both setup losses and the propagation losses of the interconnecting waveguides, which were estimated as 2 dB/cm using an optical frequency domain reflectometry^53^. A peak fiber coupling efficiency of -9 dB was achieved for a single grating at a wavelength of 1560 nm. We then changed the setup polarization to TM using the polarimeter as a reference and measure the normalized transmission spectra shown in Figure 8 (a) with a solid orange line. The same normalization used for TE is applied to TM transmission. In this case, a fiber coupling efficiency of -11.5 dB was obtained at 1560 nm (again for a single grating). Figure 8 (b) shows the difference in the fiber coupling efficiency between the TE and TM modes. A perfect polarization-independent operation is achieved at a wavelength of 1532 nm, with a 1-dB polarization insensitive bandwidth of 31 nm.

It should be noted that these results depend on both the upward diffraction efficiency $P_{\text {upward}}$ of the antenna and also on the overlap integral of the scattered field with the fiber mode, which was not considered for the design of an antenna for free-space applications. In order to better evaluate the experimental results, we hence simulate the antenna coupling efficiency with a standard single mode fiber with 3D FDTD for both polarizations. Results are reported in Figure 8 (a) with dashed lines and are in a very good agreement with the experiment. The difference between the experimental fiber transmission and the simulation prediction is smaller than 1 dB for both polarizations in the bandwidth of interest, likely due to fabrication tolerances. In the simulations, a perfect polarization insensitive coupling is obtained at a wavelength of 1538 nm, with coupling efficiency of -10.5 dB. Conservatively assuming that the additional 1 dB loss can be entirely attributed to the scattering efficiency and not to the modes overlap, allows to estimate a $P_{\text {upward}}$ of at least -4 dB (40%) for the fabricated antenna for both TE and TM modes.

## Conclusion

In this work, we proposed an innovative approach to design micro antennas with a polarization insensitive behavior based on surface grating couplers. The incorporation of an SWG metamaterial in the grating structure was the key to obtain the same effective index for TE and TM grating Bloch modes and hence the same scattering angle. The use of an L-shaped section ensured at the same time a high diffraction efficiency. As a proof-of-concept, we designed and experimentally demonstrated a polarization insensitive antenna with a compact footprint of 6.4 $\mu m$ x 2.9 $\mu m$ on a 500-nm SOI platform that successfully achieved a 1-dB polarization insensitive bandwidth of 31 nm with an estimated scattering efficiency of about -4 dB (40%).

The demonstrated antenna is compatible with standard silicon photonics fabrication processes and offers a robust solution for efficient light coupling in integrated Optical Phased Arrays regardless of the polarization state. By enabling broadband operation, the antenna holds the potential to realize next-generation photonic applications in telecommunications and beyond.

## Supplementary Information

Supplementary Information.

## Data Availability

The simulation and experimental data reported in the manuscript will be made available by the corresponding author upon reasonable request.
