# Supplementary material for: Polarization independent silicon micro antenna based on a subwavelength metamaterial
Source: Sci Rep. 2025 Apr 17;15:13276. doi: 10.1038/s41598-025-97833-3 (PMC12006522; doi:10.1038/s41598-025-97833-3)
Supplement: Supplementary file 1 — Supplementary Information. [file 41598_2025_97833_MOESM1_ESM.pdf]

Polarization independent silicon micro antenna based on a subwavelength metamaterial

Sarra Salhi[1,*,+], Xiaochen Xin[2,+], Daniel Benedikovic[2,3], Carlos Alonso-Ramos[1], Laurent Vivien[1], Delphine Marris-Morini[1], Eric Cassan[1], Winnie N.Ye[2], and Daniele Melati[1]

[1]Centre de Nanosciences et de Nanotechnologies, Université Paris-Saclay, CNRS, 91120 Palaiseau, France
[2]Department of Electronics, Carleton University, Ottawa, ON K1S 5B6, Canada
[3]Currently, with the Dept. Multimedia and Information-Communication Technology, University of Zilina, 01026 Zilina, Slovakia
[*]sarra.salhi@universite-paris-saclay.fr
[+]these authors contributed equally to this work

1. Preliminary analysis

A preliminary study was conducted to assess which of the two platforms (300 SOI and 500 nm SOI) was providing the highest diffraction efficiency for the antenna. In particular, we swept the SWG duty cycle ff_y and grating duty cycle R_L, fixing Λy = 300 nm, and we choosing Λx = 800 nm and Λx = 680 nm for the 300 nm and 500 nm platforms, respectively. As described in the manuscript, these are the largest values that allow avoiding second order diffraction. For each value of ff_y, the corresponding ff_x could be found using Figure 2. The results of 3D FDTD simulations are shown in Figure S1 for TE and TM polarized light. As can be seen, both platforms exhibit comparable diffraction efficiency for the TE mode but the 500 nm platform achieves noticeably higher efficiencies for TM. Additionally, the study allowed identifying an optimal range for R_L between 0.4 and 0.55 for further optimization.

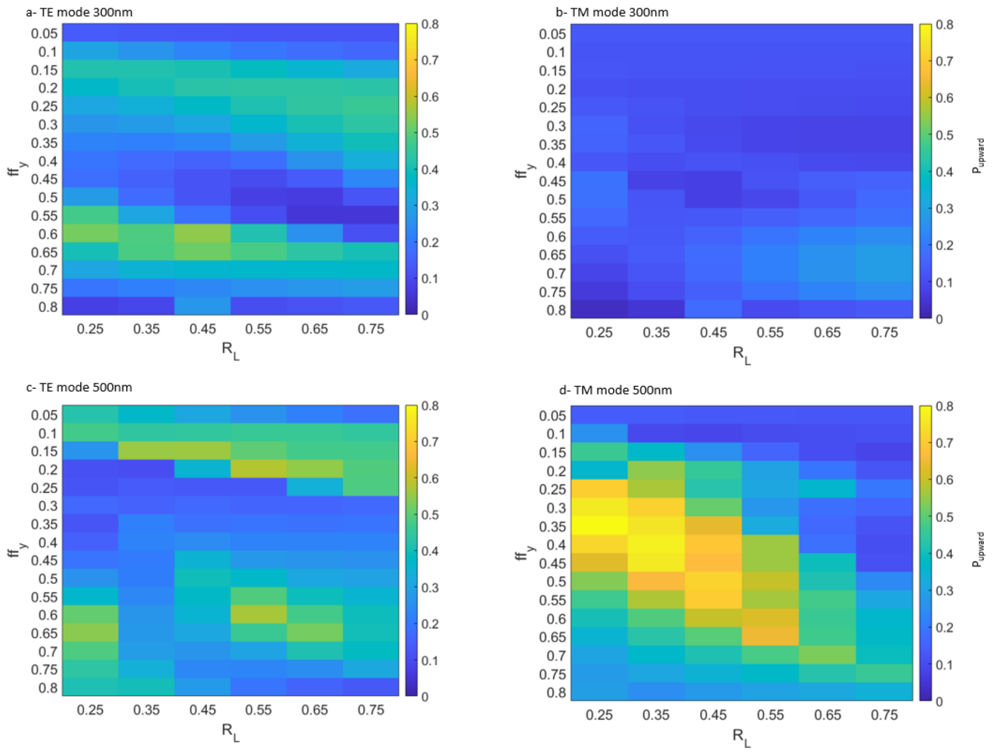

Figure S1. Diffraction efficiency P_{upward} for points along the polarization-insensitive curves (Figure 2 in the manuscript). (a) 300 nm SOI, TE mode, (b) 300 nm SOI, TM mode, (c) 500 nm SOI, TE mode, and (d) 500 nm SOI, TM mode.

2. Antenna optimization

Based on the results of the preliminary analysis, we conducted a more extensive optimization in the range for R_L values in the 0.40 – 0.55 range. For each value of R_L (0.4, 0.45, 0.5 and 0.55), sweeps of both ff_y and Λ_x were performed to find the design point that gave the highest diffraction efficiency while maintaining the radiation angle of both TE and TM identical. 3D FDTD simulations results are shown in Figures S2-S5. Based on these results, we identified the parameters that gives us the best performance for both polarizations which corresponds to R_L = 0.45, ff_y=0.6 and Λ_x =643 nm. Figure S3 is the same as Figure 3 in the manuscript.

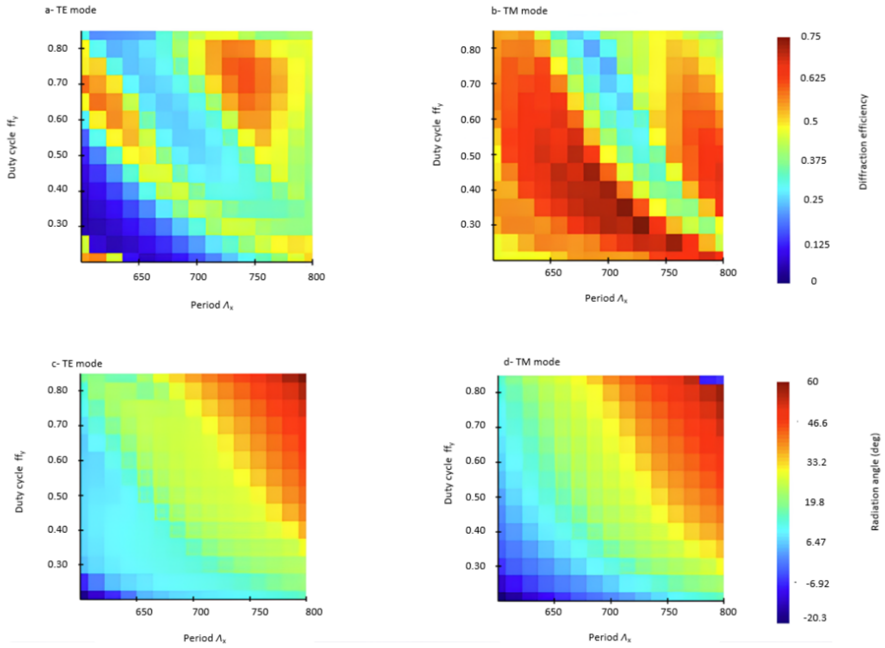

Figure S2. R_L = 0.4: diffraction efficiency (a) TE mode, (b) TM mode, and radiation angle (c) TE mode, (d)TM mode

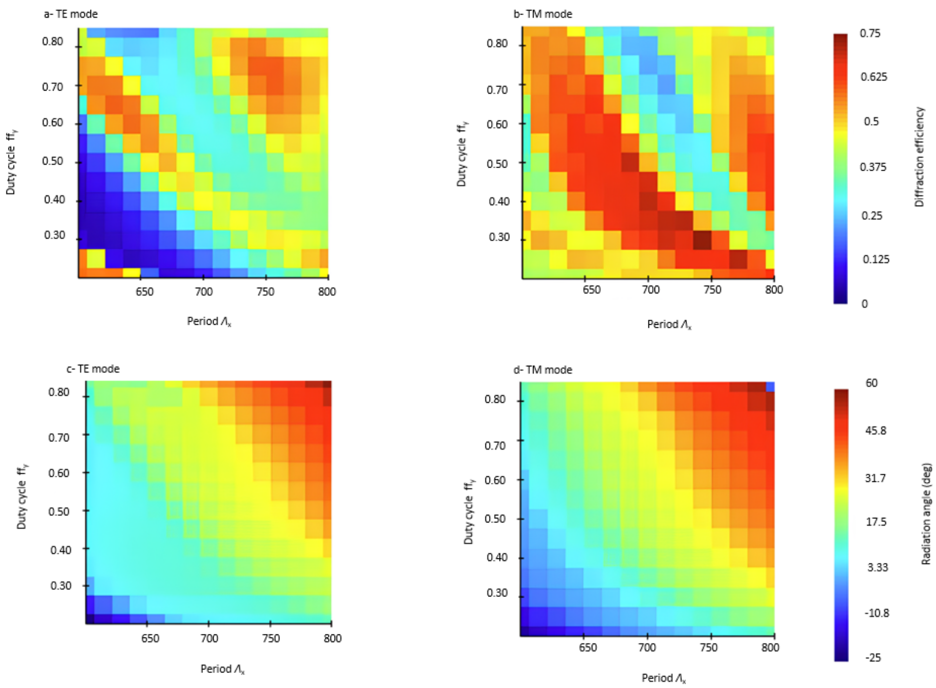

Figure S3. R_L = 0.45: diffraction efficiency (a) TE mode, (b) TM mode, and radiation angle (c) TE mode, (d)TM mode

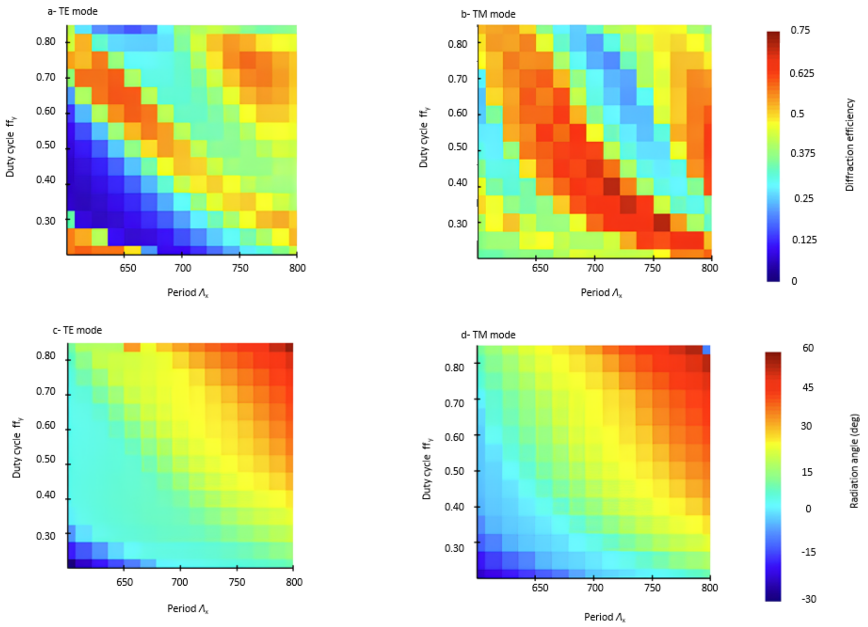

Figure 4. R_L = 0.5:diffraction efficiency (a) TE mode, (b) TM mode, and radiation angle (c) TE mode, (d)TM mode

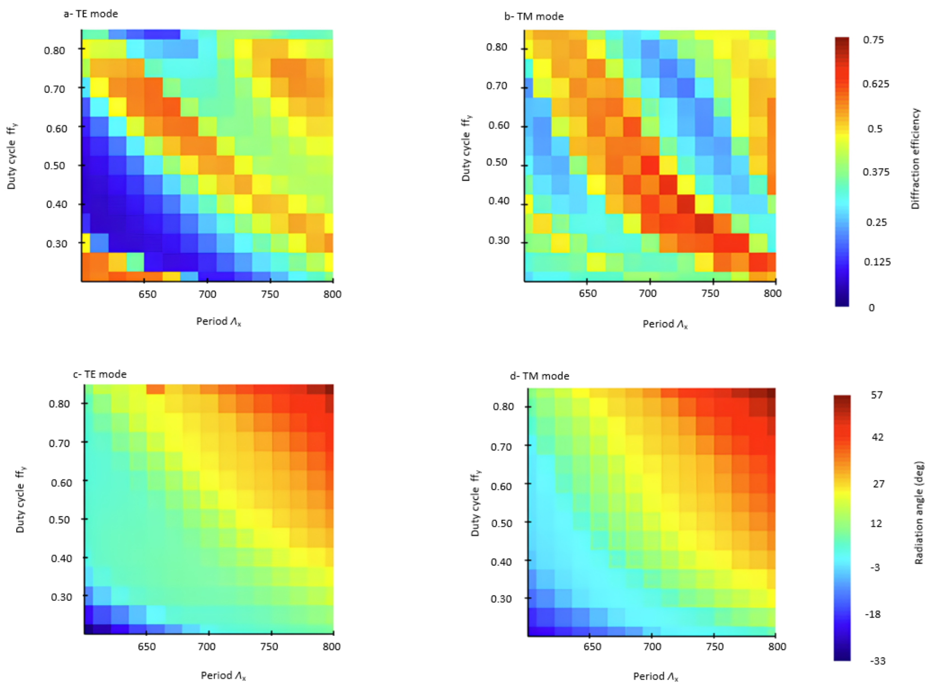

Figure 5. R_L = 0.55: diffraction efficiency (a) TE mode, (b) TM mode, and radiation angle (c) TE mode, (d)TM mode
